# Long-term effects of intrajejunal levodopa infusion on sleep in people with advanced Parkinson's disease

**DOI:** 10.3389/fneur.2023.1105650

**Published:** 2023-04-20

**Authors:** Ştefania Diaconu, Laura Irincu, Diana Ţînţ, Cristian Falup-Pecurariu

**Affiliations:** ^1^County Clinic Hospital, Braşov, Romania; ^2^Faculty of Medicine, Transilvania University, Braşov, Romania; ^3^Clinicco, Braşov, Romania

**Keywords:** intrajejunal levodopa infusion, sleep, insomnia, advanced Parkinson's disease, longitudinal

## Abstract

**Background:**

Sleep disturbances are commonly encountered in people with advanced Parkinson's disease (PD). In these stages, levodopa–carbidopa intestinal gel (LCIG) is recommended for improving motor symptoms, some non-motor dysfunctions, and quality of life in these patients. This study aimed to assess the effects of LCIG on sleep in PD in a longitudinal study.

**Study design:**

An open-label observational study in patients with advanced PD undergoing LCIG treatment was carried out.

**Measures and outcomes:**

In total, 10 consecutive advanced people with PD were evaluated at the baseline and after 6 months and 1 year, respectively, of LCIG infusion. Sleep parameters were assessed with several validated scales. We assessed the evolution of sleep parameters under LCIG infusion over time and the effects on sleep quality.

**Results:**

Significant improvement following LCIG was observed in PSQI total score (*p* = 0.007), SCOPA-SLEEP total score (*p* = 0.008), SCOPA-NS subscale (*p* = 0.007), and AIS total score (*p* = 0.001) at 6 months and 1 year, compared to the baseline. The PSQI total score at 6 months correlated significantly with the Parkinson's Disease Sleep Scale, version 2 (PDSS-2) “disturbed sleep” item at 6 months (*p* = 0.28; *R* = 0.688), while the PSQI total score at 12 months significantly correlated with the PDSS-2 total score at 1 year (*p* = 0.025, *R* = 0.697) and with the AIS total score at 1 year (*p* = 0.015, *R* = 0.739).

**Conclusion:**

LCIG infusion demonstrated beneficial effects on sleep parameters and sleep quality, which were constant over time for up to 12 months.

## 1. Introduction

Sleep disorders are common in people with Parkinson's disease (PwPD) (1). A broad spectrum of sleep complaints has been identified in these patients: insomnia, fragmented sleep, daytime sleepiness, restless legs syndrome (RLS), nocturia, and REM sleep behavior disorder (RBD) (1). Previous studies have suggested that the prevalence of sleep disturbances is higher as the disease advances (2, 3); moreover, sleep complaints are among the top five most bothersome symptoms in people with advanced PD (4) and are associated with a reduced quality of life, which is more evident as the disease progress (5).

As the co-occurrence of sleep disturbances in people with PD might have several causes, being related to complex motor complications in some of the cases, a personalized approach is advisable for the thorough assessment of sleep complaints in order to establish a comprehensive management plan (6). The diagnosis and treatment of symptoms during nighttime were reported by patients as an unmet need. More focus on the management of these symptoms with a 24-h complex approach is advised (7).

Traditional treatment in PD aims to control motor symptoms. The most commonly used oral products, levodopa/benserazide and levodopa/carbidopa, offer control of the motor features, until the onset of motor fluctuations, with no effect on disease progression (8). Levodopa–carbidopa intestinal gel (LCIG) infusion with an average duration of 15–16 h/day represents an effective treatment of complex motor symptoms in people with advanced PD (9, 10). Previous studies reported an improvement in certain non-motor symptoms, including beneficial consequences on sleep and general quality of life (11–13). These studies assessed globally the non-motor symptoms. Few studies have assessed the efficacy of LCIG over time specifically on sleep disturbances.

The objectives of this open-label observational study were to assess the effectiveness of LCIG infusion on sleep disturbances in PwPD at 6 months and 1 year after the initiation of this treatment.

## 2. Materials and methods

### 2.1. Patients and study design

Consecutive people with advanced PD who started LCIG infusion were recruited from the Department of Neurology, County Clinical Hospital of Braşov, Romania. The inclusion criteria were as follows: (i) diagnosis of PD according to the MDS clinical diagnosis criteria of PD (14); (ii) advanced stage of the disease, in treatment with LCIG (15); (iii) no dementia or severe cognitive impairment (Mini-Mental State Examination test > 24); and (iv) patients who were willing to voluntarily participate in the study (by signing the informed consent). The exclusion criteria were as follows: (i) secondary parkinsonism; (ii) advanced PD patients with indications for other device-aided therapies; (iii) severe cognitive impairment; and (iv) comorbidities known to impair sleep and quality of sleep (such as stroke, chronic pulmonary, renal, or hepatic disorders).

Patients underwent clinical assessment at the baseline (prior to LCIG treatment), and after 6 and 12 months of LCIG treatment, respectively. The evaluation of the patients was performed in “ON” states.

### 2.2. Clinical assessment

Information regarding age, gender, age at PD onset, duration of disease, Hoehn & Yahr (H&Y) stage during ON and OFF stages, previous medication for sleep disorders, and levodopa equivalent daily dose (LEDD) were collected. Patients were assessed at all three time points with validated scales for cognition evaluation (Mini-Mental State Examination—MMSE) and for sleep examination, which are described below. All patients included in the present study signed informed consent forms. The study was approved by the Ethics Committee of the University Transilvania of Braşov (1.11/01/2019).

### 2.3. Questionnaires

We used questionnaires and scales validated for PwPD. The Parkinson's Disease Sleep Scale, version 2 (PDSS-2) is a comprehensive evaluation scale of the various sleep impairments commonly identified in PD (16). For all 15 questions included in the questionnaire, the patient chooses an appropriate response from zero (never) to four (very frequent). The highest score of 60 represents maximal nocturnal disturbance, while a cutoff score of 15 was proposed to identify “bad” sleepers (16). In total, three derived PDSS-2 subscales could be identified (16), with a maximum score of 20 each: “disturbed sleep”: items 1–3, 8, and 14; “motor problems at night”: items 4–6, 12, and 13; “PD symptoms at night”: items 7, 9–11, and 15. Nocturia was assessed using item 8 of the PDSS-2.

The Scale for Outcomes in Parkinson's Disease-Sleep (SCOPA-SLEEP) (17) was designed specifically for evaluating sleep characteristics in the PD population. The subscale assessing the night symptoms (SCOPA-NS) consists of five items offering a grading possibility from 0 (“not at all”) to 3 (“a lot”), with a cutoff score of 7 indicating nighttime impairments (17, 18). Similarly, sleep symptoms during the daytime were evaluated through six items within the SCOPA-Daytime Symptoms subscale (SCOPA-DS), with a cutoff score of five, suggestive of the presence of disturbances (17). One additional item, which does not count for the total score, explores the overall sleep quality, varying from “very well” to “very bad” (17).

The Pittsburgh Sleep Quality Index (PSQI) (19) is a frequently used generic scale containing 19 questions for the assessment of the quality of sleep. A score of ≥5 was proposed to identify “bad” sleepers (19, 20).

The Athens Insomnia Scale (AIS) is a self-administered questionnaire designed for assessing the severity of insomnia. It consists of eight questions related to various aspects of insomnia, with a maximum score of 24 points indicating severe disturbances (21). A score higher than 6 is suggestive of patients with insomnia (22). We used the following criteria for grading the severity of insomnia, as proposed by Okajma et al.: absent (0–5), mild (6–9), moderate (10–15), and severe (16–24) (23).

The Epworth Sleepiness Scale (ESS) (24) was used to evaluate daily sleepiness. The patients' scores evaluated were from zero (never) to three (always), showing the probability of falling asleep in different eight daily situations. Scores higher than 10 were considered suggestive of EDS (25).

### 2.4. Data analysis

Data were analyzed using the SPSS software package for Windows, release 23.0. Descriptive data were used in order to evaluate the patients' clinical characteristics at the baseline. The Wilcoxon signed-rank test is used if the differences between pairs of data are non-normally distributed, while the Friedman test can compare more than two groups. Therefore, the Wilcoxon signed-rank test was applied for comparisons of test score values before and after the initiation of LCIG. The Friedman test was used to compare the mean ranks before and after 6 and 12 months after LCIG. We applied Bonferroni's correction in case of multiple comparisons using Microsoft Excel.

Spearman's rank correlation coefficients were employed for associations. All *p*-values reported were two-tailed. A probability *p*-value of < 0.05 was considered to be statistically significant.

## 3. Results

A total of 10 PD patients were enrolled in this longitudinal research. In the study, three patients were female subjects. The mean age ± standard deviation (SD) at inclusion was 69.8 ± 8.4 years, the mean age ± SD of disease onset was 62.8 ± 9.3 years and that of PD duration was 7 ± 2.74 years. The mean infusion duration ± SD was 15 ± 0.87 h/day. None of our patients was treated for 24 h/day. LEDD increased slightly from 1261 ± 334 mg at the baseline to 1373 ± 373 mg (*p* = 0.008) at 12 months after LCIG. None of the patients received other oral dopaminergic treatment following LCIG initiation. MAO inhibitors were withdrawn in all patients, and four patients received clonazepam for treatment of probable RBD symptoms at the baseline and during the entire follow-up period, and another patient received lorazepam for treatment of insomnia at the baseline and had reduced the dose at 12 months after LCIG implementation. The mean value of the Hoehn and Yahr stage in ON state ± SD was 3.6 ± 0.51 at the baseline, 2.9 ± 0.31 at 6 months, and 3 ± 0.47 at 12 months follow-up. Significant improvement following LCIG was observed at 6 and 12 months in the PSQI total score (*p* = 0.007), SCOPA-SLEEP total score (*p* = 0.008), SCOPA-NS subscale (*p* = 0.007), and AIS total score (*p* = 0.001; Table 1). No significant differences were observed between the ESS score (*p* = 0.27) and SCOPA-DS (*p* = 0.37) at 6 and 12 months following LCIG.

**Table 1:** Assessment of people with Parkinson's disease at LCIG infusion initiation (baseline) and after 6 and 12 months of LCIG infusion.

		**Baseline**	**6 months**	**12 months**	*p*-value^*^	*p*-value^**^
MMSE	Mean	28.9	29.10	28.90	0.568	0.999
	SD	1.52	1.28	0.99
PSQI total	Mean	10.10	6.9	6.2	**0.007**	**0.004**
	SD	3.24	2.13	2.04
SCOPA—SLEEP	Mean	15.9	10.1	9.7	**0.008**	**0.006**
	SD	5.91	2.51	2.21
SCOPA—NS	Mean	10.5	5.5	5.4	**0.007**	**0.000**
	SD	3.62	0.7	0.699
SCOPA—DS	Mean	5.4	4.6	4.3	0.37	0.369
	SD	2.98	2.31	2.31
AIS	Mean	17.1	7.4	13.7	**0.001**	**0.0001**
	SD	5.83	1.83	3.46
ESS	Mean	10.9	9.2	8.9	0.27	0.387
	SD	5.52	4.56	4.53

* Friedman test; Significant results are indicated in bold. Values are expressed as average (standard deviation).

** Bonferroni adjusted *p* < 0.016 for baseline measurement vs. 12 months. AIS, Athens Insomnia Scale; ESS, Epworth Sleepiness Scale; MMSE, Mini-Mental State Examination; PSQI, Pittsburgh Sleep Quality Index; SCOPA—NS, Scale for Outcomes in Parkinson's Disease (Sleep)—Night Symptoms; SCOPA—DS, Scale for Outcomes in Parkinson's Disease (Sleep)—Day Symptoms.

Regarding sleep quality, as evaluated with PSQI, three subdomains showed significant improvement following LCIG at 6 and 12 months (Table 2): component 1, *subjective sleep quality* (*p* = 0.004), Component 2, *sleep latency* (*p* = 0.012), and component 4, *sleep efficiency* (*p* = 0.003).

**Table 2:** PSQI test scores and its subdomains in the people with Parkinson's disease, at the baseline and after 6 and 12 months of LCIG infusion.

	**Baseline**	**6 months**	**12 months**	*p*-value^*^	*p*-value^**^
Component 1: subjective sleep quality	2.1 (0.73)	1.1 (0.31)	1.1 (0.31)	**0.004**	**0.001**
Component 2: sleep latency	2.6 (0.69)	1.9 (0.56)	1.7 (0.48)	**0.012**	**0.003**
Component 3: sleep duration	1 (1.05)	0.8 (0.42)	0.7 (0.48)	0.687	0.424
Component 4: sleep efficiency	1.3 (1.05)	0.4 (0.96)	0.3 (0.94)	**0.003**	0.039
Component 5: sleep disturbance	1.4 (0.51)	1.2 (0.42)	1.1 (0.31)	0.097	0.134
Component 6: sleep medication	0.8 (1.31)	0.5 (1.08)	0.4 (0.96)	0.156	0.448
Component 7: daytime dysfunction	0.9 (0.56)	1 (0)	0.9 (0.31)	0.717	1.000

* Friedman test; significant results are indicated in bold. Values are expressed as average (standard deviation).

** Bonferroni adjusted *p* < 0.016 for baseline measurement vs. 12 months. PSQI, Pittsburgh Sleep Quality Index.

**Figure 1:**
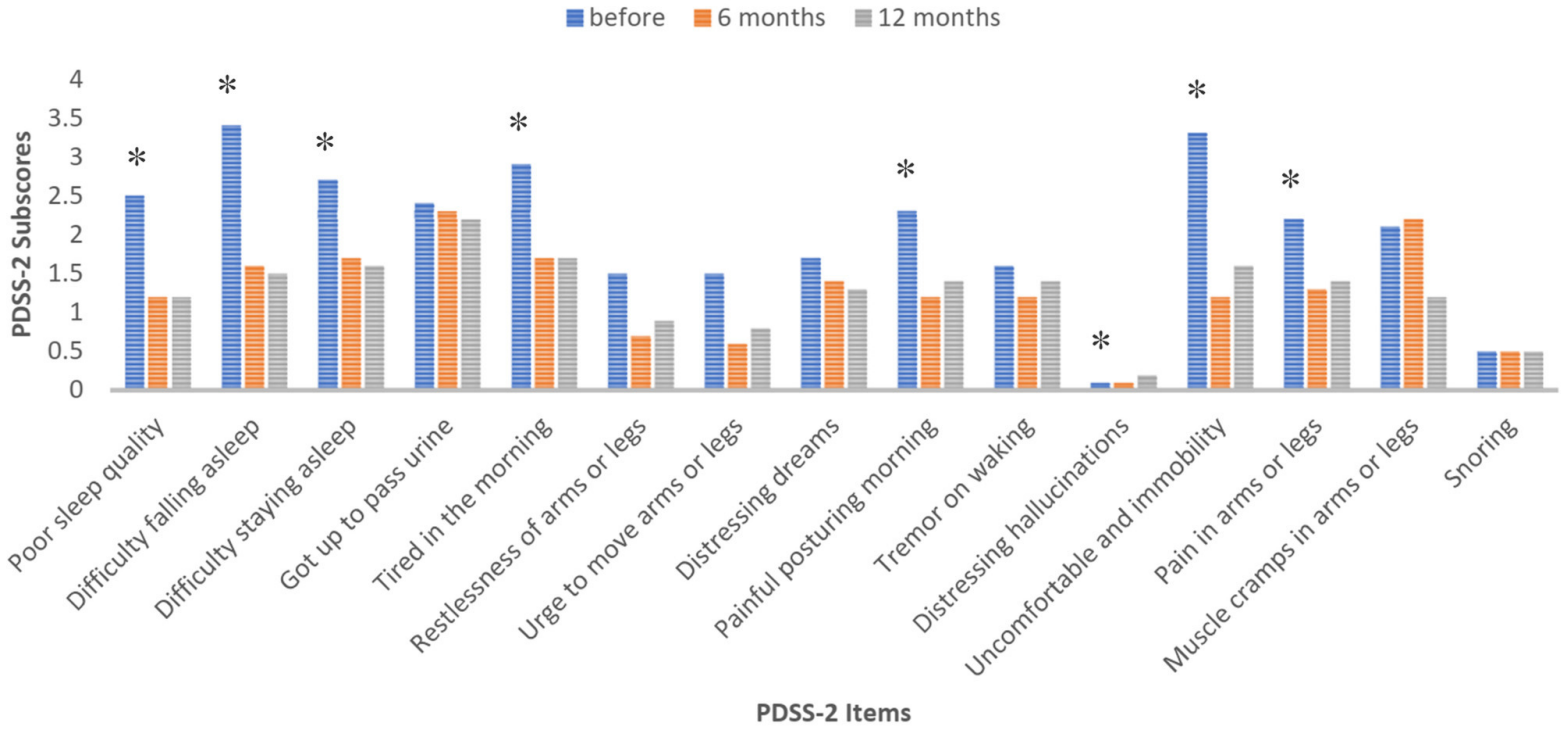
Evolution of the subscores (mean values) in the PDSS-2 at the baseline, 6 and 12 months follow-up. Values with asterisk (*) were statistically significant (*p* < 0.05), Friedman test.

Total sleep time, which was self-estimated by the patients, has increased from 5.9 ± 1.19 h before the initiation of LCIG treatment to 7 ± 0.66 h at 6 months after LCIG initiation and to 7.2 ± 0.63 h at 12 months of treatment (*p* = 0.001). As expected, the PDSS-2 total score significantly improved over time (0.006). All three subdomains of the PDSS-2 for “disturbed sleep,” “motor problems at night,” and “PD symptoms at night” showed improvement following LCIG infusion (Table 3). Specifically, the items “poor sleep quality,” “difficulties to fall asleep,” “difficulties to stay asleep,” “tiredness in the morning,” “painful postures in the morning,” “tremor when waking,” “uncomfortable and immobility,” and “painful arms or legs” demonstrated a statistically significant change from the baseline (Figure 1). There was a trend toward an improvement in symptoms related to nocturia, distressing dreams, and muscular cramps and a trend toward worsening of hallucinations; however, these trends did not reach statistical significance.

**Table 3:** PDSS-2 in the people with Parkinson's disease at the baseline and after 6 and 12 months of LCIG infusion.

	**Baseline**	**6 months**	**12 months**	*p*-value^*^	*p*-value^**^
Total sleep time (hours)	5.9 (1.19)	7.00 (0.66)	7.2 (0.63)	**0.001**	**0.007**
PDSS-2 total score	27.9 (13.08)	17.18 (6.99)	17.18 (7.6)	**0.006**	**0.003**
**Disturbed sleep**					
Item 1 reduced sleep quality	2.5 (0.84)	1.2 (0.42)	1.2 (0.42)	**0.004**	**0.004**
Item 2 difficulties to fall asleep	3.4 (1.07)	1.6 (0.51)	1.5 (0.52)	**0.003**	**0.000**
Item 3 difficulties to stay asleep	2.7 (1.05)	1.7 (0.48)	1.6 (0.51)	**0.013**	**0.008**
Item 8 passing urine during night	2.4 (1.17)	2.3 (1.15)	2.2 (1.13)	0.223	0.7
Item 14 tiredness in the morning	2.9 (1.19)	1.7 (0.67)	1.7 (0.67)	**0.016**	**0.012**
**Motor problems at night**					
Item 4 restlessness of limbs	1.5 (1.08)	0.7 (0.82)	0.9 (0.73)	0.074	0.164
Item 5 urge to move the limbs	1.5 (1.08)	0.6 (0.84)	0.8 (0.78)	0.074	0.115
Item 6 distressing dreams	1.7 (0.94)	1.4 (0.96)	1.3 (0.82)	0.156	0.327
Item 12 painful postures in the morning	2.3 (1.25)	1.2 (0.42)	1.4 (0.51)	**0.028**	0.049
Item 13 tremor when waking	1.6 (1.5)	1.2 (1.13)	1.4 (1.2)	**0.05**	0.751
**PD symptoms at night**					
Item 7 hallucinations	0.1 (0.31)	0.1 (0.31)	0.2 (0.63)	0.368	0.66
Item 9 uncomfortable and immobility	3.3 (1.05)	1.2 (0.42)	1.6 (0.51)	**0.001**	**0.000**
Item 10 painful arms or legs	2.2 (1.03)	1.3 (0.48)	1.4 (0.51)	**0.013**	0.041
Item 11 muscle cramps in limbs	2.1 (1.1)	2.2 (2.78)	1.2 (0.63)	0.065	0.037
Item 15 snoring	0.5 (0.07)	0.5 (0.07)	0.5 (0.07)	-	-

* Friedman test; Significant results are indicated in bold. Values are expressed as average (standard deviation).

** Bonferroni adjusted *p* < 0.016 for baseline measurement vs. 12 months. PD, Parkinson's disease; PDSS-2, Parkinson's Disease Sleep Scale version 2.

With regard to SCOPA-SLEEP total and SCOPA-NS subscores, results showed a quite remarkable improvement in sleep quality. Mean SCOPA-SLEEP total was 15.9 ± 5.91 at the baseline, with an improvement after 6 months (10.1 ± 2.51), respectively after 12 months (5.91 ± 2.21; *p* = 0.008; Table 1). The evolution of the overall item (“C1—Overall, how well you slept at night during the past month?”) in SCOPA-SLEEP is presented in Table 4. A trend of improvement in subjective quality of sleep was noted in most of the patients after LCIG therapy at 6 and 12 months.

**Table 4:** Evolution of overall item (C1—“Overall, how well you slept at night during the past month?”) in SCOPA-SLEEP. Scores are shown in percentages.

**Overall, how well you slept at night during the past month?**	**Baseline (%)**	**6 months follow up (%)**	**12 months follow up (%)**
Very well	0	0	0
Well	0	50	40
Rather well	20	30	50
Not well but not badly	10	20	0
Rather badly	30	0	10
Badly	30	0	0
Very badly	10	0	0

SCOPA-SLEEP, Scale for Outcomes in Parkinson's Disease.

Regarding the quality of sleep and other sleep parameters, Spearman rank correlation analyses were performed. The PSQI total score at 6 months correlated significantly with PDSS2 “disturbed sleep” item at 6 months (*p* = 0.28; R = 0.688); moreover, there was a significant correlation between the PSQI total score at 12 months and the PDSS-2 total score at 12 months (*p* = 0.025, *R* = 0.697) and between PSQI total score at 12 months and AIS total score at 12 months (*p* = 0.015, *R* = 0.739). Significant differences were also found for the baseline measurement vs. 12 months reevaluation, after Bonferroni correction, except for Component 4: Sleep efficiency (*p* 0.003 vs. 0.039) of PDSS2.

## 4. Discussion

This study evaluated the effect of LCIG treatment in advanced PD at 6 and 12 months. Sleep complaints have been reported by more than half of PD patients, being known to significantly affect the quality of life in these patients (26). Therefore, it is essential for clinicians to establish a comprehensive therapeutical plan that should be effective for both motor and non-motor characteristics. Sleep disorders were commonly reported in our study group. In total, four patients were previously diagnosed with probable RBD and were treated with clonazepam, one patient was treated with lorazepam for persistent insomnia, while the other patients had no treatment for their sleep disorders.

Several explanations for the development of sleep disturbances in advanced PD have been proposed. Due to fluctuations in the motor symptoms and the association of painful cramps, akinesia, and dystonia during night, sleep might also be disturbed (27). Neurodegeneration of the regions involved in sleep regulation and low concentrations of dopaminergic medications during night might be involved in the occurrence of sleep complaints in advanced PD (28). Moreover, early morning off periods, which can be identified in almost 60% of PwPD with dopaminergic treatment, might also cause several disturbances during nighttime, given the number of non-motor symptoms that can be associated—the urgency of urination, anxiety, pain, and limb paresthesia (29).

The main finding of our research was a significant improvement in sleep parameters and quality of sleep at 6 and 12 months after LCIG initiation. Total scores of the PDSS-2, SCOPA-SLEEP and SCOPA-SLEEP NS subscale, AIS, and PSQI showed significant reductions compared to the baseline. Overall sleep disturbances persisted at 12-month follow-up but with reduced intensity.

Insomnia, one of the most frequently declared sleep problems in PD patients, was demonstrated to be less severe with LCIG therapy (most of the subjects reported moderate insomnia at the baseline and mild insomnia at the two follow-up visits, as recorded with the total AIS score). The items from the PDSS-2 concerning insomnia (“difficulty falling asleep” and “difficulty staying asleep”) showed significant changes from the baseline, suggesting improvements in this domain. Poor sleep quality, tiredness in the morning, as well as other symptoms related to motor problems or PD symptoms at nighttime (painful postures in the morning, tremor, immobility and discomfort, and limb pain) presented a significant improvement with continuous dopamine delivery, as evaluated with PDSS-2. On the contrary, for item 7 of the PDSS-2 (“distressing hallucinations”), it was noticed a slight worsening at 12 months compared to that of the baseline and 6 months, and item 15 (“snoring”) was constant during the entire follow-up period. Daytime sleepiness (as evaluated with ESS and SCOPA-DS) did not show statistically significant improvement during the follow-up period. This result might be explained either by the small sample size or by increased LEDD administration.

To the best of our knowledge, few studies have characterized the evolution of sleep parameters over time as a primary outcome in patients treated with LCIG infusion.

Our conclusions are in line with the results of other studies, which revealed that an appropriate dopaminergic supply during nighttime can be effective on sleep complaints. However, most of the previous questionnaire-based studies have assessed the effectiveness of LCIG infusion using multidomain scales such as the Non-Motor Symptoms Questionnaire (NMSQ) or Non-Motor Symptoms Scale (NMSS), which contain only some items related to sleep complaints. In our study, we used several validated scales to examine more specifically insomnia, sleep fragmentation, EDS, and the consequences of sleep disturbances on quality of life.

One of the first studies that demonstrated the benefits of LCIG on sleep was conducted by Honig et al. and included 22 PD patients who were followed up for 6 months (30). Significant improvements were observed in nine domains of the NMSS, including the sleep/fatigue domain (30).

Buongiorno et al. observed a significant decrease in insomnia at 3 months following the initiation of LCIG infusion, using a semi-structured interview (31).

One multicentric study analyzing the GLORIA registry included 258 PD patients in treatment with LCIG infusion, over a period of 24 months (12). The sleep domain was assessed with the NMSS, and at the last follow-up visit, a significant reduction in the sleep/fatigue domain score was recorded, compared to the baseline. The improvement in sleep quality was consistent with the alleviation of motor symptoms (12). Similar results were obtained by Chaudhuri et al. (13) from the GLORIA database. Among other non-motor symptoms, the sleep/fatigue domain assessed with the NMSS showed significant improvement compared to the baseline and also a significant association between the NMSS sleep scores and the improvement in the quality of life was observed (13).

The results at 1-year follow-up in the DUOGLOBE study showed an improvement in sleep complaints, as evaluated with the total PDSS-2 score. Improvements were also noticed in daytime sleepiness (ESS total score) (32); our results were in line with this even though in our study the positive effect on daytime symptoms measured with ESS and SCOPA-DS did not reach statistical significance.

Sleep parameters were assessed using PDSS-2 in two other previous studies (33, 34), with a significant improvement in LCIG therapy over time. Zibetti et al. (33) have also noticed that daytime sleepiness improved after 2–4 months of LCIG treatment in 12 patients.

On the contrary, De Fabregues et al. demonstrated that sleep quality was still poor in patients undergoing LCIG infusion, but it did not get worsened by the end of the follow-up period (35).

A better quality of sleep was observed in the study group. PSQI total scores decreased progressively at 6 and 12 months. Moreover, most of the patients included in our study evaluated a shorter sleep latency and better sleep efficiency (calculated as estimated total hours asleep/total hours in bed). Subjective improvement in sleep quality was correlated at 6 months with the alleviation of disturbed symptoms at night (difficulty initiating and maintaining sleep, nocturia, and tiredness in the morning) and at 12 months with an overall improvement in sleep complaints and reduced insomnia symptoms.

The effectiveness of LCIG infusion on sleep parameters and quality of sleep may be explained by a more stable concentration of levodopa obtained with the constant substance administration even though the infusion was stopped during nighttime. There are data suggesting that daytime LCIG may significantly improve sleep quality, as assessed using the PDSS scale and Parkinson's disease questionnaire PDQ-8 (27). The improvement of motor symptoms which appears to be steadier following continuous drug administration than oral therapies can be reflected on sleep patterns and might contribute to subjective improvements of complaints related to sleep.

We are aware that the present study has several limitations. The main limitation of this study is the lack of a control group. This was a questionnaire-based study, and the sleep disturbances identified in our patients were not assessed by objective methods, such as polysomnography. Several parameters, such as total sleep time, were self-estimated by the patients (anamnestic and not measured by polysomnography). We did not include the opinion or the evaluation of caregivers concerning the sleep quality of the patients. Furthermore, this is an unblinded open-label study. Considering the small sample size of our study, larger trial studies addressing specifically the effect of LCIG on sleep may be conducted. The power of the study is low; thus, some no significant differences in our study can become significant in a larger group. Several sleep disturbances, such as RLS, sleep apnea, or RBD were not evaluated specifically.

In conclusion, our findings suggest that sustained, long-term improvements in sleep parameters and sleep quality were obtained in patients undergoing LCIG infusion. In busy clinical practice, when complex objective assessments such as actigraphy or polysomnography are time-consuming and/or expensive to use, scales and questionnaires might be useful to monitor the effectiveness of medication on sleep. One may consider using the PDSS-2 to follow up on sleep disturbances under LCIG infusion as it assesses various aspects of sleep, is brief, and is easy to administer. Sleep disturbances have an important impact on quality of life, along with other motor and non-motor features associated with advanced PD. In these complex cases, the concept of personalized medicine should be applied. Our data support the observation that proper management of the motor symptoms with continuous levodopa delivery may also confer better sleep and a better quality of life for this category of PD patients.

## Data availability statement

The raw data supporting the conclusions of this article will be made available by the authors, without undue reservation.

## Ethics statement

The studies involving human participants were reviewed and approved by the Ethics Committee of the University Transilvania of Braşov (1.11/01/2019). The patients/participants provided their written informed consent to participate in this study.

## Author contributions

ŞD, CF-P, and LI worked on conception and design of the study, and data collection, and analysis and interpretation of data. ŞD was drafting the article. LI, DŢ, and CF-P revised the article for important intellectual content. ŞD, LI, DŢ, and CF-P gave final approval of the version to be submitted. All authors contributed to the article and approved the submitted version.
